# The Effect of Meteorological Factors on the COVID-19 Pandemic in Northeast Turkiye

**DOI:** 10.7759/cureus.36934

**Published:** 2023-03-30

**Authors:** Gürkan Altuntas, Murat Cetin, Mustafa Emin Canakci, Mümin Murat Yazıcı

**Affiliations:** 1 Emergency Department, Recep Tayyip Erdoğan University Training and Research Hospital, Rize, TUR; 2 Emergency Department, Manisa Merkezefendi State Hospital, Manisa, TUR; 3 Emergency Department, Eskisehir Osmangazi University, Eskisehir, TUR

**Keywords:** environmental factors, emergency department, covid-19, pandemic, meteorological factors

## Abstract

Introduction

Although various studies have been conducted on the relationship between meteorological factors and coronavirus disease 2019 (COVID-19), this issue has not been sufficiently clarified. In particular, there are a limited number of studies on the course of COVID-19 in the warmer-humidity seasons.

Methods

Patients presenting to the emergency departments of health institutions and to clinics set aside for cases of suspected COVID-19 in the province of Rize between 1 June and 31 August 2021 and who met the case definition based on the Turkish COVID-19 epidemiological guideline were included in this retrospective study. The effect of meteorological factors on case numbers throughout the study was investigated.

Results

During the study period, 80,490 tests were performed on patients presenting to emergency departments and clinics dedicated to patients with suspected COVID-19. The total case number was 16,270, with a median daily number of 64 (range 43-328). The total number of deaths was 103, with a median daily figure of 1.00 (range 0.00-1.25). According to the Poisson distribution analysis, it is found that the number of cases tended to increase at temperatures between 20.8 and 27.2°C.

Conclusion

It is predicted that the number of COVID-19 cases will not decrease with the increase in temperature in temperate regions with high rainfall. Therefore, unlike influenza, there may not be seasonal variation in the prevalence of COVID-19. The requisite measures should be adopted in health systems and hospitals to manage increases in case numbers associated with changes in meteorological factors.

## Introduction

Health systems and hospitals have had to adapt to new conditions during the coronavirus disease 2019 (COVID-19) pandemic in order to cope with the outbreak and maintain the provision of basic health services [1]. As seen in the COVID-19 pandemic, hospital emergency departments in particular are at the forefront of the fight against viral respiratory disease outbreaks [2].

Components such as the infectious agent and host and environmental factors generally affect the epidemiology of infectious diseases. Understanding the effect of these factors on the pandemic is an important component in the control, prevention, and management of patient density in hospitals [3]. Predicting which meteorological conditions may exacerbate the airborne disease and transmission burden during viral respiratory disease outbreaks may assist in the taking of the requisite precautions against potential rises in patient density in hospitals.

In addition to human-to-human transmission, meteorological factors are also thought to affect the vitality, transmission, and spread of viruses. Several infectious diseases may be affected by such environmental factors as humidity, temperature, and population density [4,5]. Human respiratory pathogens (such as influenza) generally exhibit a seasonal spread, with incidence increasing in winter and decreasing in summer. However, despite this information, it is difficult to predict whether COVID-19 will decrease or disappear in warmer seasons. Although the relationship between meteorological factors and COVID-19 is unclear, various epidemiological studies have revealed such an association [6-10]. Although many studies have been published on this subject, the relationship between meteorological factors and COVID-19 is not clear enough. However, the number of reports on the impact of weather factors such as temperature and humidity on the spread of the virus in warm and rainy tropical countries is still limited [11].

This study investigated the effect of meteorological factors on the number of cases and hospital admissions due to the COVID-19 pandemic in the summer season. Our goal was to provide scientific evidence on the spread of COVID-19 depending on meteorological factors.

## Materials and methods

The research was conducted as a retrospective study. Following receipt of Clinical Research Ethics Committee of Recep Tayyip Erdogan University Faculty of Medicine ethical committee approval (ethics committee date and no: 2022/130), patients presenting to the emergency departments of health institutions and clinics dedicated to suspected COVID-19 patients in the province of Rize, Turkey, between 1 June and 31 August, 2021, and meeting the case definition in the Turkish COVID-19 epidemiological guideline were included in the study. The diagnosis of COVID-19 was confirmed by positive reverse transcription-polymerase chain reaction (RT-PCR) testing of nasopharyngeal swabs. Total case numbers in the province during the study period were recorded on a daily basis, together with numbers of hospitalizations, admissions to intensive care, and deaths.

The study group consisted of patients admitted to emergency departments of health institutions and clinics with suspicion of COVID-19 in Rize province of Turkey, who met the case definition in the COVID-19 epidemiological guidelines and whose COVID-19 diagnosis was confirmed by positive reverse transcription-PCR (RT-PCR) testing of nasopharyngeal swab samples.

Daily Atmospheric Pressure (hPa), Mean Daily Relative Humidity (%), Mean Daily Wind Speed (m÷s), Mean Daily Temperature (°C), and Total Daily Rainfall (mm=kg÷m²) during the study period were also recorded. The association between meteorological data and case numbers was investigated from these recorded data. Data including meteorological findings were obtained from the Rize Provincial Meteorology Directorate.

Statistical analysis

Continuous data were expressed as mean ± standard deviation or median (interquartile range) values depending on the normality of distribution. Categorical data were expressed as percentage (%) values. The Shapiro Wilk test was applied to determine normality of data distribution. The Mann-Whitney U test was applied in the comparison of two non-normally distributed groups, and the Student’s t test in the case of normal distribution. Pearson’s chi-square and Pearson’s Exact chi-square analyses were used in the evaluation of the cross-tables created. The positive distribution test was applied to determine the presence of disease and Poisson regression analysis was employed to identify factors affecting distribution. Statistical analyses were performed on SPSS Statistics software, Version 21.0 (IBM Corp. Armonk, NY). A p value<0.05 was regarded as statistically significant.

## Results

A total of 80,490 tests were performed on patients presenting to emergency departments and clinics dedicated to individuals with suspected COVID-19 during the study period. The daily median number of tests was 643 (501-1278). The total case number was 16270, with a daily median number of 64 (43-328). The total death rate was 103, with a daily median value of 1.00 (0.00-1.25). Mean daily actual pressure was 1011±3.44 kPa, the mean daily relative humidity was 79.9±4.95%, and the median daily wind speed was 0.900 (0.775-1.000) m/s. The median daily temperature was 24.1 (21.9-25.3) degrees. Rainfall was present on 49 (53.3%) of these days. A comparison of rainy and rain-free days is shown in Table 1. No difference was observed between rainy and rain-free days in terms of case numbers. Relative humidity was higher on rainy days.

**Table 1:** Analysis in Terms of Rainy and Non-Rainy Days IQR: Interquartile Range * Mann Whitney U Test, ** Student-t Test

	Rain (n=49)	No rain (n=43)	p
Test numbers (IQR)	695 (514-1445)	613 (493-1120)	0.215*
Case numbers (IQR)	73 (47-388)	60 (40.5-182)	0.129*
Death count (IQR)	1 (0-2)	1 (0-1)	0.700*
Daily pressure kPa± SD	1012 ± 3.43	1010±3.25	0.017**
Daily relative humidity % ± SD	81.6 ± 5.45	78.0 ±3.48	<0.001**
Daily wind speed m/s, (IQR)	0.900 (0.800-1.000)	0.900 (0.750-1.000)	0.333*
Daily temperature ^o^C, (IQR)	23.3 (21.1-24.3)	25.0 (23.5-26.0)	<0.001*

An increase was observed in case and fatality numbers as the number of tests rose. In addition, test numbers were also higher on days when the wind speed was lower. Factors affecting test numbers according to Poisson distribution are shown in Table 2. An increase in case numbers presenting to hospitals was observed on days of high temperature and relative humidity. Temperatures on days of high case numbers ranged between 20.8 and 27.2 °C. Factors affecting case numbers according to Poisson distribution are shown in Table 3.

**Table 2:** Factors Affecting Test Numbers According to Poisson Distribution

	Odds Ratio	95% Confidence Interval	P
Case number	1.004	1.004-1.004	<0.001
Death count	1.036	1.028-1.045	<0.001
Daily pressure kPa	0.996	0.991-1.000	0.040
Daily relative, humidity %	0.997	0.994-0.999	0.010
Daily wind speed m/s	0.878	0.839-0.920	<0.001
Daily temperature ^o^C	1.001	0.995-1.008	0.717

**Table 3:** Factors Affecting Case Numbers According to Poisson Distribution

	Odds Ratio	95% Confidence Interval	P
Test number	1.001	1.001-1.001	<0.001
Death count	0.989	0.976-1.002	0.103
Daily pressure kPa	0.995	0.988-1.001	0.116
Daily relative humidity %	1.022	1.018-1.027	<0.001
Daily wind speed m/s	1.026	0.935-1.125	0.594
Daily temperature ^o^C	1.040	1.029-1.052	<0.001

## Discussion

Health system policymakers must develop sustainable policies concerning the spread of cases, and especially regarding climate change and improvement, and implement plans to encourage strategies to reduce air pollution in order to prevent the adverse effects of future pandemics on public health and economic systems. Heterogeneous findings have been reported following analysis and research into the effects of meteorological variables on the COVID-19 pandemic. However, some studies have reported a negative correlation between temperature and the incidence of COVID-19 [12,13]. One study reported a negative correlation between both temperature and humidity and new daily COVID-19 cases worldwide [6] and showed that the effect of weather conditions on the spread of COVID-19 might be a global one. Research from China also reported a negative association between temperature and humidity and COVID-19 [14]. These studies have observed a decrease in daily case numbers and deaths caused by COVID-19 in countries with high temperatures and humidity (hot countries). 

However, Raza et al. reported a positive association between COVID-19 cases and temperature. Similarly to the present study, Raza et al. showed that a 1°C increase in mean temperature in Pakistan resulted in a 0.024-fold increase in the expected log number of COVID-19 cases at Poisson regression [9]. During winter, temperature and humidity are low in temperate regions. Viruses are more easily transmitted through aerosols at low temperatures than through direct or indirect contact. However, aerosol transmission is reduced in the rainy seasons of hot and rainy regions, but transmission by direct contact is more common. Although high temperatures can reduce the stability of the virus and its level in the viral aerosol, the amount of virus deposited on surfaces may increase as the temperature increases [15,16].

Rize is one of the provinces of Turkiye with the highest rainfall. Rainfall in Rize is equally balanced in all seasons, and there is no dry season. In the study published by Auler et al., they said that contrary to many studies, the conclusion that high temperature and high humidity reduce the transmission of COVID-19 does not reflect the results in tropical regions with heavy rainfall [11]. For the study period selected, they mentioned, the transmission of COVID-19 did not decrease at temperatures above 21°C in some cities [11]. In our study, COVID-19 cases were seen on warmer and humid days in the summer season. Hongjing et al. said that in some countries, high temperatures or high relative humidity might also increase the risk of COVID-19 infection [10]. Xie and Zhu found no evidence to support that COVID-19 case numbers could decline when the weather becomes warmer [17]. Additionally, based on data collected from various regions, Tan et al. reported that the optimal environmental temperature associated with SARS cases was between 16 °C and 28 °C [18]. He et al. stated that although high temperatures are thought to be associated with a lower probability of virus transmission, it is unlikely that the pandemic will decrease in numbers during the warmer seasons [16].

Transmission via droplets and aerosols is important for the spread of viruses. Taking precautions against the transmission of viruses transmitted through the air, including SARS-CoV-2, is of very great importance. Factors such as adverse weather conditions, the high variability of viral loads, and inefficient ventilation can increase the spread of viruses [19]. Measures should be taken to reduce airborne transmission in order to avoid any increase in the prevalence of local infection prevalence and to lower the rate of transmission in risky weather conditions or climatic changes [20]. Understanding the effect of meteorological factors on COVID-19 infection in the light of available data represents an important step in combatting this disease. This can also serve as a guide for national health providers and for the effective use of health resources in case of potential epidemics. 

There are a number of limitations to this study. The first is that the research was conducted in a city in the northern region of Turkiye. The study was also performed in the summer, and a broader analysis might be performed with data from other seasons. Second, due to the infectious nature of COVID-19, it may be necessary also to examine other factors involved in the transmission. More data may be required in order to perform a comprehensive study, since transmission is affected by many variables, including population density, social distancing, the resilience of the inhabitants, socio-cultural levels, and personal hygiene measures.

## Conclusions

According to the findings of this study, it can be said that the number of COVID-19 cases will not decrease in hot seasons. In hot-humid regions with heavy rainfall, the number of COVID-19 cases may not decrease with the warming of the weather. Thus, unlike the flu, the prevalence of COVID-19 may not vary seasonally, and the pandemic is unlikely to decrease in numbers during the warmer seasons. Potential increases in patient numbers during epidemics can be predicted with the assistance of meteorological factors. However, the impact of meteorological factors on the transmission of COVID-19 may be weaker compared to factors such as virus mutations, vaccination, social distancing. We think that it will be useful to take appropriate measures in advance to predict the potential increase in patient numbers that may occur, adopt the necessary precautions in hospitals, and manage the increasing workload.
